# Musculoskeletal ultrasonography combined with electromyography in the diagnosis of massage-inducted lateral plantar nerve injury: A case report

**DOI:** 10.1097/MD.0000000000021130

**Published:** 2020-07-10

**Authors:** Zhende Jiang, Hanyang Zhang, Tong Yu, Yanhui Du, Zhihui Qian, Fei Chang

**Affiliations:** a Key Laboratory of Bionic Engineering, Jilin University; b Orthopaedic Medical Center, The Second Hospital of Jilin University, Changchun, Jilin Province, China.

**Keywords:** electromyography, lateral plantar nerve, massage, ultrasound

## Abstract

**Introduction:**

It is well known that foot massage is a very prevalent stress relief method in China. Literatures have reported various massage-inducted peripheral nerve injuries. However, massage-inducted lateral plantar nerve (LPN) injury is very rare. Here, we represent an unusual case of massage-inducted LPN damage, and we also report the diagnostic method of this patient using musculoskeletal ultrasonography combined with electromyography (EMG).

**Patient concerns:**

A 21-year-old woman presented symptoms of redness, swelling, pain and numbness in the medial right ankle joint for 2 days.

**Diagnosis:**

The results of musculoskeletal ultrasonography and EMG provide great help for doctors to make accurate diagnosis. The patient was eventually diagnosed with LPN injury.

**Interventions:**

No further foot massage was allowed. Vitamin B12 was taken orally for 2 months. Conservative therapy, including electrical stimulation therapy and infrared therapy, was conducted. Besides, active rehabilitation training was also performed.

**Outcomes:**

The discomfort symptoms were relieved significantly after 2 months conservative treatment. Clinical symptoms and EMG examination illustrated satisfactory result during follow up time.

**Conclusion:**

The report showed that the masseur should be very careful when doing foot massage to prevent nerve damage. Besides, musculoskeletal ultrasonography combined with EMG can provide important evidence for accurate and effective diagnosis of LPN injury.

## 1 Introduction

It is well known that foot massage is a very prevalent stress relief method in China. In literature, numerous massage-inducted neuropathy have been described, including sciatic nerve injury,^[1]^ cervical cord injury,^[2,3]^ brachial plexus injury,^[4]^ radial nerve injury,^[5]^ posterior interosseous nerve injury,^[6,7]^ recurrent motor branch of median nerve injury,^[8]^ spinal accessory nerve injury.^[9]^ Other complications resulting from massage therapy include renal artery embolization,^[10]^ ureteral stent displacement,^[11]^ hepatic hematoma,^[12]^ retinal and cerebral artery embolism,^[13]^ hematomas, pulmonary emboli, rupture of the uterus, and ulceration with infection.^[14]^ However, no cases of massage-inducted lateral plantar nerve (LPN) compromise have been reported, to our knowledge.

The etiologies of LPN injury include local traction and compression, blunt trauma, fracture, neuritis, mass lesions and puncture wounds.^[15]^ However, there was no LPN injury caused by foot massage in literatures. Routine examination, including X-ray, computed tomography and magnetic resonance imaging, is difficult to evaluate nerve injury, and often leads to missed diagnosis in clinical practice. We combined musculoskeletal ultrasonography and electromyography (EMG) to diagnose LPN injury. In this case report, we represent an unusual patient of massage-induced LPN injury. Furthermore, we also share our experience of diagnostic methods and therapeutic strategies.

## 2 Ethic

This case report was approved by the institutional review board of the second hospital of Jilin university. Informed written consent was obtained from the patient for publication of this case report and accompanying images.

## 3 Case report

**Figure 1:**
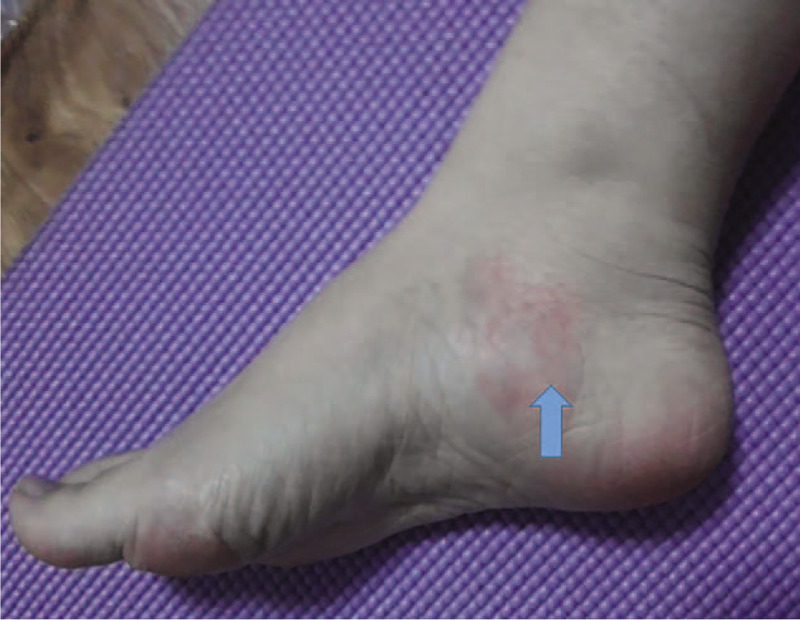
Clinical picture showed that the skin of the medial right ankle was redness and swelling.

A 21-year old female patient presented with right foot local pain, redness, swelling **(**Fig. 1**)** and heel pain with numbness for 2 days. There was history of foot massage 2 days ago. Clinical examination showed a positive Tinel sign upon compression of the local skin.

**Figure 2:**
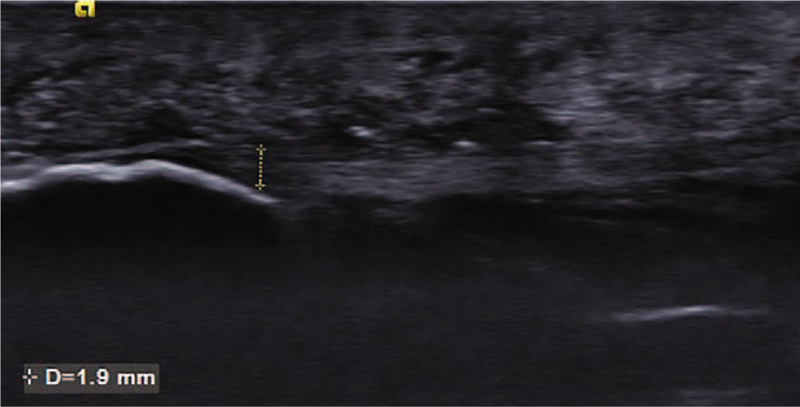
Musculoskeletal ultrasound showed that both the thickness of plantar fascia and the fibrillar structure were normal.

**Figure 3:**
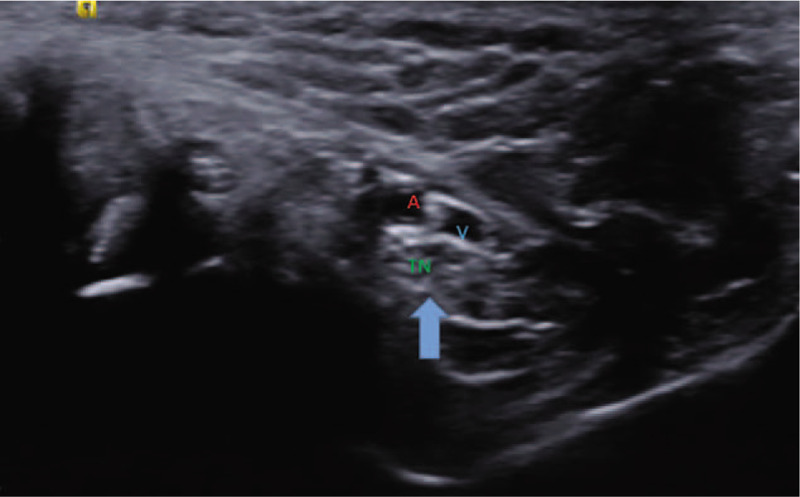
Tibial nerve (TN) at the level of ankle. A represents artery and V presents vein.

**Figure 4:**
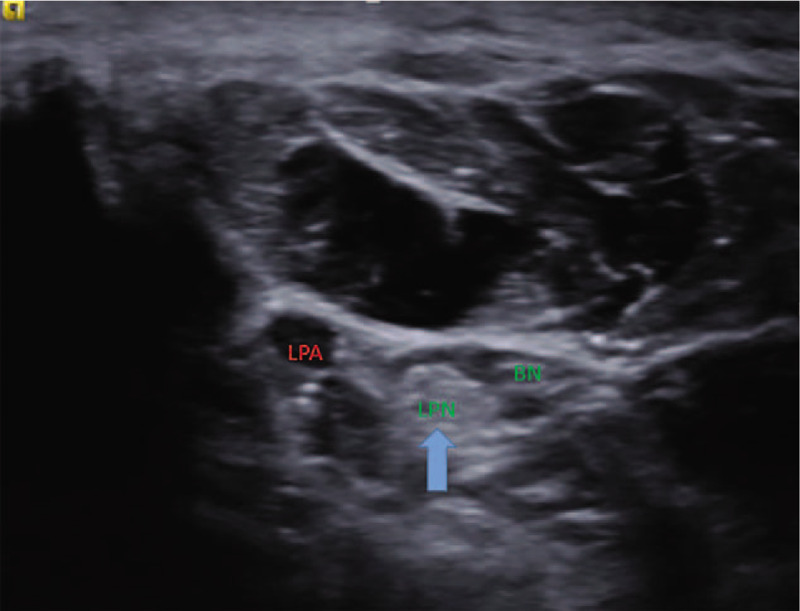
The branches of tibial nerve, including the lateral plantar nerve (LPN) and the Baxter's nerve (BN). LPA represents lateral plantar artery and the vein was not shown due to compression.

**Figure 5:**
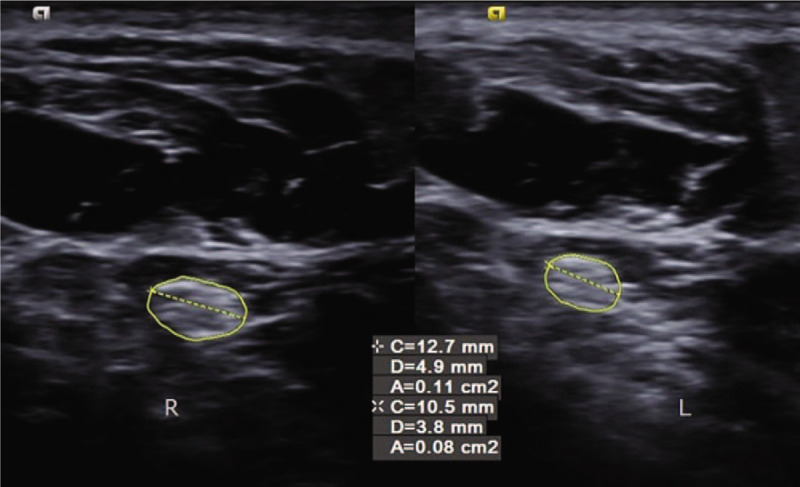
The CSAs of right and left LPN were 11 mm^2^ and 8 mm^2^ respectively, the length diameter on the cross section of right and left LPN was 4.9 mm and 3.8 mm. CSA = cross sectional area, LPA = lateral plantar nerve.

**Figure 6:**
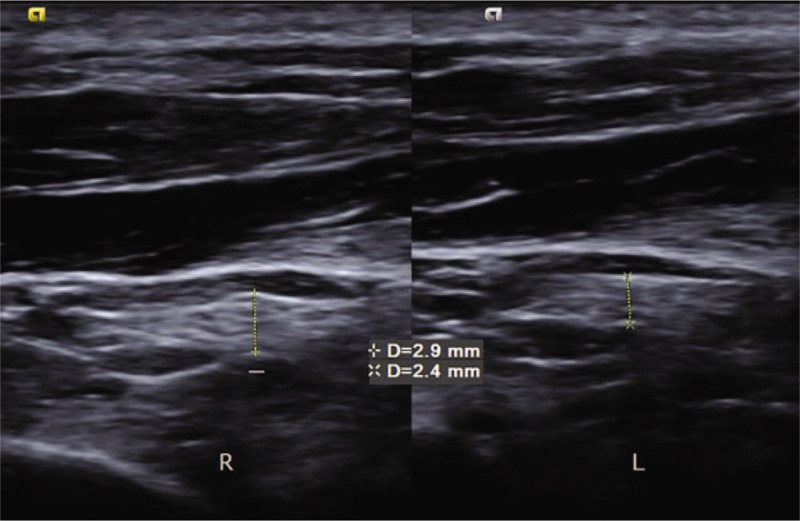
The thickness on the longitudinal section was 2.9 mm in right foot and 2.4 mm in left foot.

Musculoskeletal ultrasonography (SIEMENS, S2000 Helx) with a high frequency probe of 18 MHz was selected to detect plantar fascia and tibial nerve. Musculoskeletal ultrasound showed that both the thickness of plantar fascia **(**Fig. 2**)** and fibrous structure were normal. Doppler showed no perifascial effusion, calcification or congestion. Therefore, plantar fasciitis was excluded. It is difficult to locate the branches of tibial nerve directly under musculoskeletal ultrasound. Firstly, we confirmed tibial nerve at the level of right ankle **(**Fig. 3**)**. Secondly, we scan the tibial nerve distally to the affected site. The branches of tibial nerve, include LPN and Baxter's nerve^[16]^**(**Fig. 4**)**, were detected. Thirdly, by continuing to scan distally, we found that there were perifascial collections around right LPN and increased cross sectional area (CSA) of right LPN **(**Fig. 5**)**. The cross sections and longitudinal sections on left side were also measured and showed normal results **(**Figs. 5 and 6).

EMG (Endeavor CR, Natus Neurology Incorporated, United States) measurements were performed on both lower extremities, and the result showed that the sensory nerve conduction velocity of right LPN was evoked difficulty. However, the sensory nerve conduction velocity of left LPN was normal, and the conduction velocity was 52 m/s.

No further foot massage was allowed. Vitamin B12 was taken orally for 2 months. Conservative therapy, includes electrical stimulation therapy and infrared therapy, was conducted. Besides, active rehabilitation training was performed.

The discomfort symptoms were relieved significantly after 2 months conservative treatment. Clinical symptoms, musculoskeletal ultrasonography and EMG illustrated satisfactory results during the 2 years follow up time.

**Figure 7:**
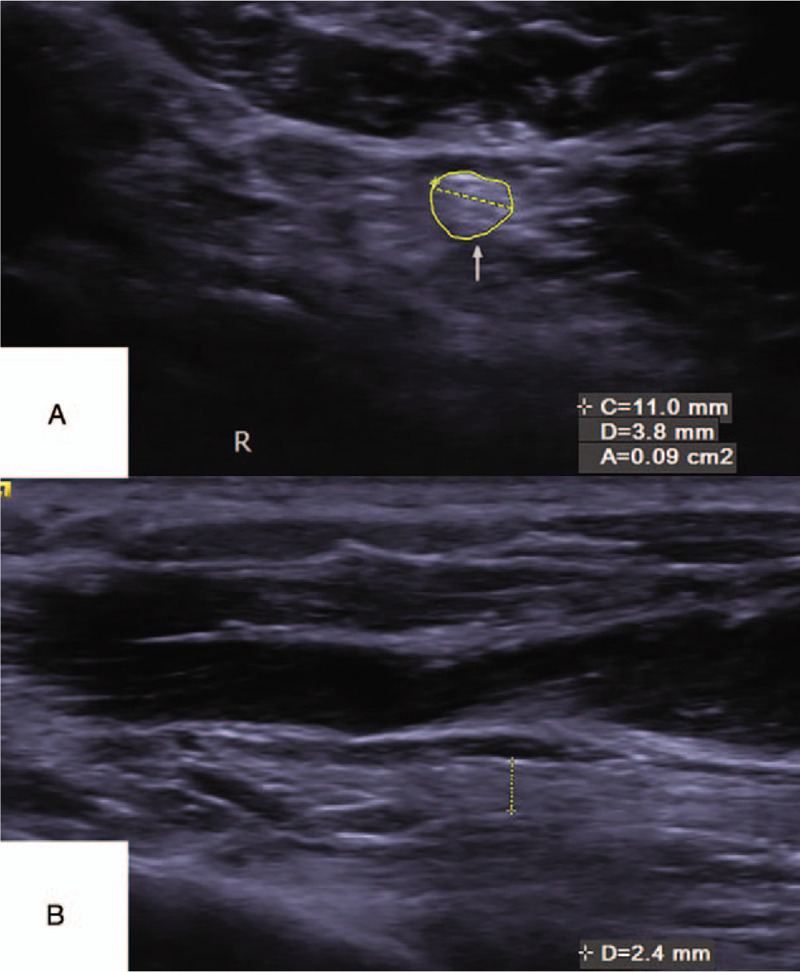
The results of musculoskeletal ultrasonography after 2 mo conservative treatment. The CSA of right LPN was 9 mm^2^, the length diameter on the cross section of the LPN was 3.8 mm (A), and the thickness of the longitudinal section was 2.4 mm (B). CSA = cross sectional area, LPA = lateral plantar nerve.

The CSA of right LPN was improved from 11 mm^2^ to 9 mm^2^, the length diameter on the cross section of right LPN was decreased from 4.9 to 3.8 mm, and the thickness of the longitudinal section was improved from 2.9 to 2.4 mm **(**Fig. 7A-B**)**. All results are close to the left foot, which CSA was 8 mm^2^, length diameter on the cross section was 3.8 mm, and the thickness of longitudinal section was 2.4 mm.

**Figure 8:**
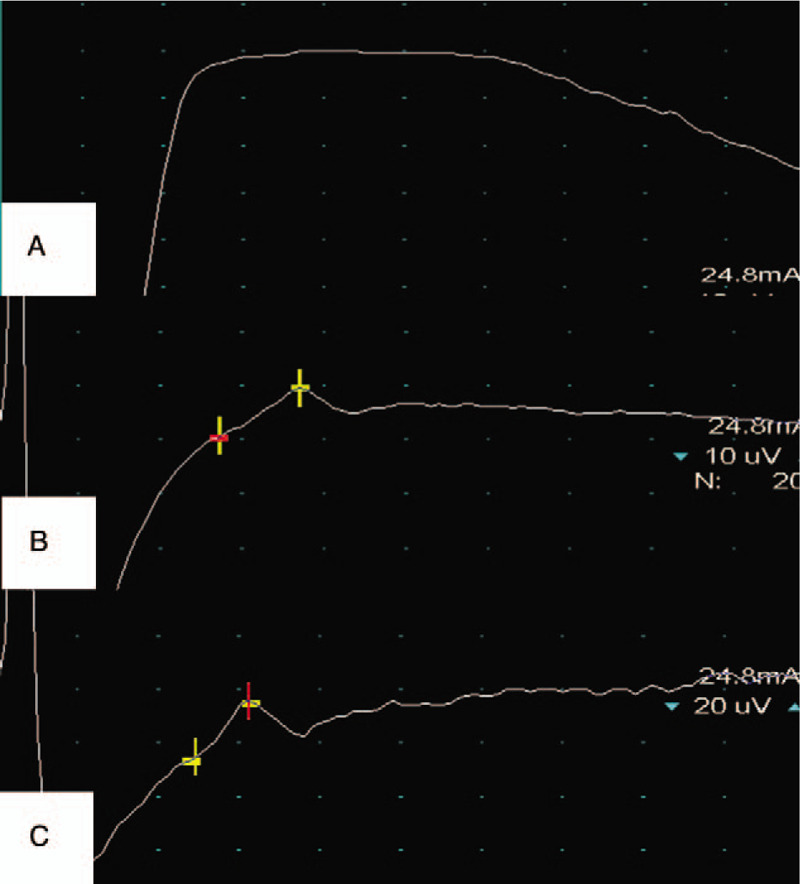
The EMGs of right (A) and left (B) lower extremity before treatment and after treatment (C). EMG = electromyography.

Re-examination of EMG showed that the sensory nerve conduction velocity of right LPN was improved from 0 to 51m/s **(**Fig. 8A-C**)**.

## 4 Discussion

Previous literatures have reported various massage-inducted complications,^[1,4,5,12]^ including peripheral nerve injuries and other structures damage. However, to date, no foot massage-induced neuropathy has been reported, especially massage-inducted LPN injury is very rare. In this report, we represent an unusual patient of LPN injury that result from foot massage. Furthermore, we also share our experience of diagnostic methods and therapeutic strategies.

Thorough clinical assessment, ultrasound and EMG examination are the critical diagnostic evidence of massage-induced nerve injury.^[5]^ Through detailed clinical evaluation, we can know whether nerves are compressed during massage. Musculoskeletal ultrasonography evaluation can directly observe whether there is edema around the injured nerve, compression of effusion, and the size of the diameter of the injured nerve. EMG assessment act as a key role in estimating the lesion localization and degree of the nerve injury.^[17]^ In this case, medication and rehabilitation were applied for the treatment of nerve injury, and satisfied result was obtained. We attribute this positive result to the accurate diagnosis of ultrasound combined with electromyography and proper conservative treatment.

LPN is a branch of the tibial nerve that travels in the deep side of the hallux abductor muscle and then obliquely anteriorly to the superficial side of flexor digitorum longus and quadratus digitorum. LPN sends out superficial and deep branches, which control the contraction of muscles of flexor digitorum brevis, medial interosseous muscles, etc. Small cutaneous branches of LPN dominate the sensation of the lateral plantar skin before the formation of superficial and deep branches. In this case, only sensory impairment occurred after the massage, and we initially judged that the small cutaneous branches of LPN might be damaged.

Musculoskeletal ultrasonography has the advantage of direct observation of the nerves and surrounding tissues.^[18]^ LPN can be detected by an ultrasound probe, and we compared the cross-sectional diameters, echogenicity and vascularity of LPN on both feet. In this study, the transverse diameter of LPN in the affected side was significantly larger than that in the healthy side, which provided important evidence for diagnosis nerve injury. Colleagues claimed^[18]^ that ultrasound is considered to be a convenient and rapid diagnostic tool for radial neuropathy, and we think this is also suitable for rapid detection of LPN damage.

EMG is an important means to diagnose neuropathy. It can locate nerve lesions, evaluate the severity of nerve injury and judge the prognosis. The injured nerve is shown on electromyogram as slow nerve conduction and denervation of its innervating muscle. In this case, EMG results showed that the lateral plantar sensory nerve were both conductive block and decreased in amplitude on the massage side but well reproduced on non-massage therapy side. The findings were in keeping with a poorly functioning right LPN and would fit with a history of nerve damage.

In a review of the literature, the treatment strategies for neuropathy are medication, rehabilitation, perineural steroid injection and surgical repair.^[1,4]^ Usually the prognosis of massage-induced neuropathy is satisfactory. Chang et al^[4]^ reported that massage-induced brachial plexus injury was completely recovered after conservative treatment. Hsu et al^[5]^ described that acute radial neuropathy at the spiral groove following massage was achieved a near normal recovery after conservative treatment. Arnold et al^[19]^ reported a satisfactory prognosis of acute compressive radial neuropathy in 51 patients. This suggested to us that conservative treatment, including medications for pain control and rehabilitation for muscle strength recovery, were useful strategies for massage-induced nerve injury. Hsu et al^[5]^ reported that if the discomfort symptoms do not relieve after 3 to 6 months conservation treatment, then surgical intervention should be carried out. In our case, a near total recovery was achieved after the 2 months conservative treatment.

## 5 Conclusion

The report showed that the masseur should be very careful when doing foot massage to prevent nerve damage. Besides, musculoskeletal ultrasonography combined with EMG can provide important evidence for accurate and effective diagnosis of LPN injury.

## Author contributions

**Conceptualization:** Zhende Jiang.

**Data curation:** Zhende Jiang, Tong Yu.

**Formal analysis:** Zhende Jiang, Yanhui Du.

**Methodology:** Hanyang Zhang.

**Supervision:** Zhihui Qian, Fei Chang.

**Writing – original draft:** Zhende Jiang, Hanyang Zhang, Tong Yu, Yanhui Du.

**Writing – review & editing:** Zhihui Qian, Fei Chang.
